# Models for short term malaria prediction in Sri Lanka

**DOI:** 10.1186/1475-2875-7-76

**Published:** 2008-05-06

**Authors:** Olivier JT Briët, Penelope Vounatsou, Dissanayake M Gunawardena, Gawrie NL Galappaththy, Priyanie H Amerasinghe

**Affiliations:** 1 International Water Management Institute, P.O. Box 2075, Colombo, Sri Lanka; 2 Swiss Tropical Institute, Socinstrasse 57, P.O. Box CH-4002, Basel, Switzerland; 3 US Agency for International Development, P.O. Box 7856, Kampala, Uganda; 4 Anti Malaria Campaign, Head Office Colombo, Sri Lanka; 5 International Water Management Institute Sub Regional Office for South Asia, c/o ICRISAT, Patancheru, AP 502 324, Andhra Pradesh, India

## Abstract

**Background:**

Malaria in Sri Lanka is unstable and fluctuates in intensity both spatially and temporally. Although the case counts are dwindling at present, given the past history of resurgence of outbreaks despite effective control measures, the control programmes have to stay prepared. The availability of long time series of monitored/diagnosed malaria cases allows for the study of forecasting models, with an aim to developing a forecasting system which could assist in the efficient allocation of resources for malaria control.

**Methods:**

Exponentially weighted moving average models, autoregressive integrated moving average (ARIMA) models with seasonal components, and seasonal multiplicative autoregressive integrated moving average (SARIMA) models were compared on monthly time series of district malaria cases for their ability to predict the number of malaria cases one to four months ahead. The addition of covariates such as the number of malaria cases in neighbouring districts or rainfall were assessed for their ability to improve prediction of selected (seasonal) ARIMA models.

**Results:**

The best model for forecasting and the forecasting error varied strongly among the districts. The addition of rainfall as a covariate improved prediction of selected (seasonal) ARIMA models modestly in some districts but worsened prediction in other districts. Improvement by adding rainfall was more frequent at larger forecasting horizons.

**Conclusion:**

Heterogeneity of patterns of malaria in Sri Lanka requires regionally specific prediction models. Prediction error was large at a minimum of 22% (for one of the districts) for one month ahead predictions. The modest improvement made in short term prediction by adding rainfall as a covariate to these prediction models may not be sufficient to merit investing in a forecasting system for which rainfall data are routinely processed.

## Background

Malaria has been a major public health problem in Sri Lanka [1] until recently. Since the year 2000, incidence has dwindled [2] with only 591 reported cases for 2006 [3]. It is unstable and fluctuates in intensity both spatially and temporally, thus resources for control have to be spread in time and space to be prepared for outbreaks, which have occurred in the past despite very aggressive and effective malaria control operations [4]. Having a forecasting system in place will contribute to a more focussed approach for control, and have a positive impact on the resource allocation for malaria control over space and time. This paper explores different models for malaria case prediction, which is possible due to the availability of long, dense and reliable records of malaria cases and climate variables in Sri Lanka [5].

While many factors play a role in the spatial and temporal distribution of malaria, climate variability (both spatial variation of the long term seasonal mean of weather variables, and temporal aberrations from the long term seasonal mean) has been shown to be important in explaining its occurrence [6-8] and is considered a major determinant [9]. Temperature, rainfall, and humidity affect breeding and survival of a certain (sub) species of anopheline mosquitoes that carry the malaria parasite, as well as development of malaria parasites within vector mosquitoes, thereby creating a link between weather and malaria.

At present, there are no practical tools for temporal prediction of the occurrence of malaria based on observed rainfall or weather forecasts in Asia, although these are in development [10]. For Africa, such tools have been developed [11] and applied [12]. Recent work [13,14] focuses on malaria early warning systems, in which flags are raised when epidemics are expected. Setting the threshold for what is an epidemic (defined as a number of cases substantially exceeding that what is expected based on recent experience or what is thought normal) is subjective. The term epidemic does not combine well with the term prediction (if the expected number is predicted based on recent experience, the prediction can never be 'epidemic' according to the above definition). It is difficult to define, especially in Sri Lanka, at what level malaria incidence is thought to be normal, as the malaria time series show strong long-term fluctuations and it is, therefore, difficult to set thresholds. In general, disease forecasting is most useful to health services when it predicts case numbers two to six months ahead, allowing tactical responses to be made when disease risk is predicted to increase (or decrease) [15]. For this reason this paper avoids the problem of setting epidemic thresholds, and focuses on forecasting malaria cases.

Malaria case numbers are influenced by factors intrinsic to malaria such as infectivity, immunity and susceptibility of vectors and humans, and extrinsic, environmental factors such as rainfall. The number of possible models for malaria prediction is infinite. In biological process models, typically consisting of sets of equations, prediction can be done with details of all pathways, parameters and variables believed to be important for the dynamics of the disease [15]. In statistical models, temporal or spatial autoregressive terms account for the fact that case numbers depend on past or nearby case numbers through (sometimes cyclical) intrinsic processes, as well as for (unobserved) extrinsic auto correlated factors or factors with fading effects. This study was limited to some statistical models that are relatively easily implemented (without taking into consideration complex biological processes and their parameters), and/or that have been successful elsewhere in malaria forecasting studies. With sufficient temporal autocorrelation in malaria case time series, malaria cases can be predicted based on previous values [16]. However, predictions from statistical models are made under the assumption that the relationships established based on past observations remain the same in the future. Therefore, statistical models require experience with as wide a spectrum of conditions as possible. In this light, the present low case numbers, have been unprecedented in the time series under study, and a caution should be in place. More complex statistical models can be constructed where malaria incidence in an area is, apart from its own previous values, also dependent on (previous) values in neighbouring areas, or covariates such as rainfall [17,18]. These latter models require more inputs and therefore more resource intensive to apply, particularly where covariate data need to be acquired and processed in a timely manner to be useful for forecasting. In this paper, it was examined which standard time series statistical model would be useful for forecasting malaria, and it was examined whether addition of rainfall to autoregressive models could improve malaria prediction in districts with one to four month forecasting horizons.

## Methods

This section describes the data used for the analyses, methods for pre-processing of the data, types of models tested and the criteria for model selection.

### Malaria data

The count of blood films examined for malaria as well as those positive for malaria per month reported by government health facilities and aggregated by medical officer of health (MOH) area (which represent sub district health administrative divisions) were provided by the Anti Malaria Campaign of Sri Lanka for the period 1972 – 2003. In addition, data aggregated by district were available for the years 2004 – 2005. For some of the records, the number of blood films examined was marked as "not received" (and therefore classified as missing). For 14.90% of the MOH area level records, the value was zero, or left blank. For the latter records, there was ambiguity as to whether the data value could be missing due to problems in data recording, or genuinely zero if no patients presented themselves for examination in that particular area in that particular month. As such, in a data cleaning procedure (see section on statistical methods), 1.4% of the records was declared as not available (NA) if the number of blood films examined was marked as "not received" (0.95%), or if the number of blood films could be classified as a lower additive outlier (0.44%). The data from districts in the north and east, where data gathering and reporting was affected by the armed conflict, had the largest percentage of data labelled not available: Jaffna (5.4%), Mannar (26.1%), Vavuniya (8.9%), Kilinochchi (2%), Trincomalee (2%) and Ampara (5.4%). After imputation, MOH area level data for positive cases were aggregated to district resolution and combined with the district level data (for the period 2004 – 005).

### Rainfall data

**Figure 1:**
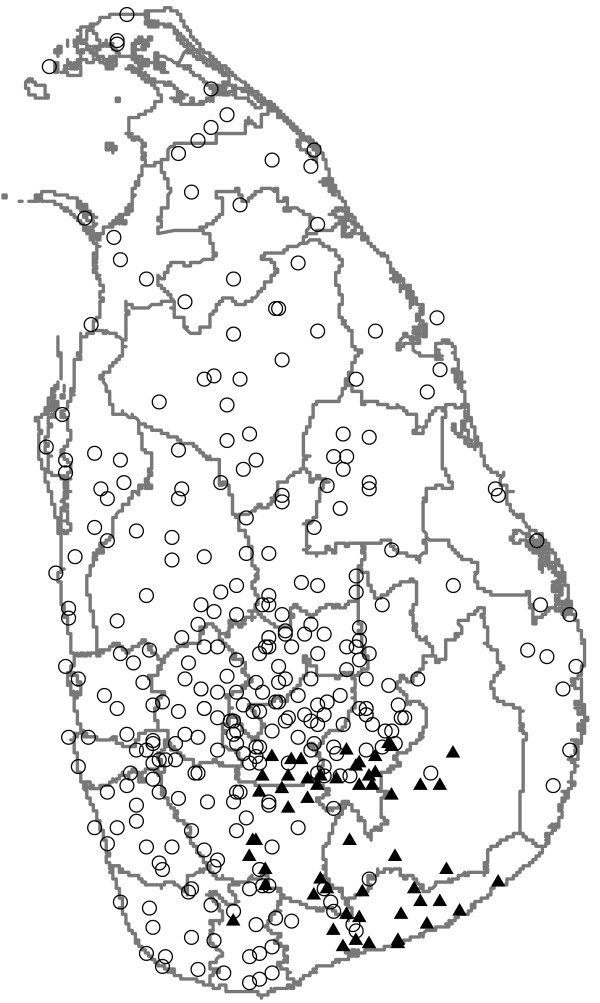
**Rainfall stations**. Location of stations measuring rainfall for which monthly data (open circles) and daily data (solid triangles) were available. Grey lines represent current boundaries of the 25 districts. The time period for which data was available varied per station.

Records of precipitation (rain fall) collected by 342 stations across the island were purchased from the Meteorological Department of Sri Lanka (see Figure 1). This consisted mostly of monthly aggregate data, but for an area in the south (Ruhuna), daily rainfall data were also available for 57 stations covering partly the districts of Ratnapura, Hambantota, Badulla and Moneragala, for the period January 1972 – March 2003. Three stations with consistently aberrant rainfall, detected through cross validation using kriging [19], were removed from the dataset. Monthly rainfall surfaces were created through spatial prediction using kriging [19]. From the daily data available, the monthly "rainy day index" was calculated for each station by dividing the number of days per month that rainfall was larger than zero by the number of days that a reading for rainfall was available. Monthly rainy day index surfaces were generated following the same procedure as for the total monthly rainfall. From each monthly rainfall surface, the average value of rainfall/rainy day index was extracted for each district.

### Statistical methods

The monthly count of malaria positive blood slides in each district *y*_*t *_were transformed to normality via the logarithmic transformation *z*_*t *_= log (*y*_*t *_+ 1). The models tested included exponentially smoothing and auto-regressive integrated moving average (ARIMA) models [20]. As some of the district malaria count time series showed strong seasonality, seasonality was also modelled. In models using exponential smoothing, seasonality was included using the Holt-Winters procedure [20]. In ARIMA models, seasonality was included via three different approaches which are all widely used in literature: seasonality through fixed (monthly) effects; seasonality through harmonics; and through random effects using seasonal mixed auto-regressive integrated moving average (SARIMA) models. Whether or not covariates such as rainfall and concurrent malaria case counts in neighbouring areas improved the predictive ability of the models was also tested. In addition, the seasonal adjustment method used by Abeku and colleagues [16], was tested.

### Exponentially weighted moving average models

The additive Holt-Winters prediction function (for time series with period length *s*) at time *t*+*h *is given by the following equation:

$${\hat{z}}_{t+h}=m_{t,h}+S_{t,h}$$

where *m*_*t*,*h *_is the average number of cases at time *t*+*h *expressed as a trend *r*_*t*,*h *_and an overall mean term *a*_*t*_, that is *m*_*t*,*h *_= *r*_*t*,*h *_+ *a*_*t*_. *S*_*t*,*h *_is a seasonality term at time t+h, such that *S*_*t*,*h *_= *S*_*t*-*s*+1+(*h*-1)mod *s *_where (*h *- 1) mod *s *is the remainder of *h*-1 after division by *s *(*e.g*. 14mod12 = 2). Thus

(1) $${\hat{z}}_{t+h}=a_{t}+hr_{t}+S_{t-s+1+(h-1)\mathrm{mod}s}$$

where *a*_*t*_, *r*_*t *_and *S*_*t *_are calculated by the following recursive functions:

*a*_*t *_= *α*(*z*_*t *_- *S*_*t*-*s*_) + (1 - *α*)(*a*_*t*-1 _+ *r*_*t*-1_);

*r*_*t *_= *β*(*a*_*t *_- *a*_*t*-1_) + (1 - *β*)*r*_*t*-1_;

*S*_*t *_= *γ*(*z*_*t *_- *a*_*t*_) + (1 - *γ*)*S*_*t*-*s*_.

Both seasonal and non-seasonal (with *γ *fixed to 0) models were tested using the function "HoltWinters" in the package "stats" of the statistical software package "R".

### (S)ARIMA regression models

It was assumed that *z*_*t *_is Gaussian distributed, *z*_*t *_~ *N *(*μ*_*t*_, *σ*^2^), with mean *μ*_*t *_and variance *σ*^2^. Further, it was assumed that

(2) *μ*_*t *_= *f*(*z*_*t*_, *d*, *p*, *x*_*t*_) + *g*(*u*_*t*_, *q*)

where *f*(*z*_*t*_, *d*, *p*, *x*_*t*_) and *g*(*u*_*t*_, *q*) model the temporal correlation as

*f*(*z*_*t*_, *d*, *p*, *x*_*t*_) = Φ_*p*_(*B*)(1 - *B*)^*d*^(*x*_*t *_- *z*_*t*_) + *z*_*t *_and *g*(*u*_*t*_, *q*) = Θ_*q*_(*B*)*u*_*t *_- *u*_*t*_

where

Φ_*p*_(*B*) = 1 - *φ*_1_*B *- ... - *φ*_*p*_*B*^*p*^;

Θ_*q*_(*B*) = 1 - *θ*_1_*B *- ... - *θ*_*q*_*B*^*q*^;

*u*_*t *_is Gaussian white noise;

*x*_*t *_= *m*_*t *_+ *S*_*t*_;

*S*_*t *_models the seasonal process;

*m*_*t *_models the mean of *z*_*t*_

*B *is a backshift operator with *B*^*d*^(*z*_*t*_) = *z*_*t*-*d*_.

The seasonality in the ARIMA models of equation 2 was modelled by fixed effects. In particular it was assumed:

• *S*_*t *_= 0 (A non seasonal model),

• $\begin{array}{ccc} S_{t}=\sum_{}^{12}\left(\alpha_{k}\delta_{k,t}\right) & \text{where} & \delta_{k,t}=\{\begin{array}{ll} 1 & \text{if t}=\text{nk}\\ 0 & \text{if t}\neq \text{nk} \end{array} \end{array}$

(Seasonality through fixed effects for months: Note that in this model *m*_*t *_does not contain an intercept to avoid over parameterisation),

• $S_{t}=\sum_{i}^{2}A_{i}\sin(2\pi f_{i}t+2\pi \phi_{i})$

a second order harmonic component where *A*_*i *_is the amplitude of harmonic *i*; *f*_*i *_is the frequency of harmonic *i*, with *f*_1 _= 1/*s*, *f*_2 _= 2/*s*; and *ϕ*_*i *_is the phase shift (in units of time) of harmonic *i*.

Also, a multiplicative seasonal ARIMA(p,d,q)*(P,D,Q) model (henceforth SARIMA) was considered with period *s*, obtained by modifying equation 2 into

*μ *= *f*(*z*_*t*_, *d*, *p*, *D*, *P*, *s*, *m*_*t*_) + *g*(*u*_*t*_, *q*, *Q*, *s*)

where

$$f(z_{t},d,p,D,P,s,m_{t})=\Phi_{p}(B){\left(1-B\right)}^{d}{\left(1-B^{s}\right)}^{D}\Phi_{P}^{*}(B^{s})(m_{t}-z_{t})+z_{t};$$

$$g(u_{t},q,Q,s)=\Theta_{q}(B)\Theta_{Q}^{*}(B^{s})u_{t}-t_{t};$$

$$\Phi_{P}^{*}(B^{s})=1-\varphi_{1}^{*}B^{s}-\ldots -\varphi_{P}^{*}B^{sP};$$

$$\Theta_{Q}^{*}(B^{s})=1-\theta_{1}^{*}B^{s}-\ldots -\theta_{Q}^{*}B^{sQ};$$

and Φ_*p*_(*B*), Θ_*q*_(*B*), *u*_*t*_, *m*_*t *_and *B *as explained above.

The function "arima" in the package "stats" of the statistical software "R" was used to calculate the prediction criterion. Tested models included all (Gaussian) ARIMA models possible with combinations of parameters (*p*, *d*, *q*) with *p*, *q *∈ {0,1,2} and with *d *= 1, without explanatory variables, and all (Gaussian) SARIMA models possible with combinations of parameters (*p*, *d*, *q*, *P*, *D*, *Q*) with *p*, *q *∈ {0,1,2} and *d *= 1 and *P*, *D*, *Q *∈ {0,1}, also without explanatory variables. An intercept was not included in the mean as it drops out of the equation due to differencing (*d *= 1). The differencing also removes effects of trends such as potentially caused by population growth.

Covariates were included in the term *m*_*t*_. In particular, 1) $m_{t}=\sum_{j}\beta_{j}z_{j,t-1}$ where *z*_*j*,*t*-1 _is the transformed malaria count at month *t *- 1 in neighbour *j*; 2) *m*_*t *_= *βχ*_*t*-*l *_where *χ*_*t *_is the rainfall parameter in month *t*-*l *with *l *= lag. Rainfall was considered at lags of one to four months preceding malaria and in the following forms: untransformed monthly rainfall, logarithmically transformed monthly rainfall, rainy day index (for those districts appropriate), monthly rainfall factored into quintiles (in case of non-linear relationships), and rainfall with a separate coefficient for each of the twelve months, *i.e*. a coefficient for January rainfall, one for February, etc., in order to allow for seasonally varying effects. For each district, covariates were tested by including them into the (S)ARIMA model that performed best for the respective district and lag.

### Estimation of non-available malaria count data

In a data cleaning procedure, the time series of blood film counts in MOH areas were logarithmically transformed to normality (after the value one was added to the data). Under the null hypothesis, each observation was assumed to be part of a seasonal autoregressive integrated moving average (SARIMA) process with parameters *p *= 0, *d *= 1, *q *= 1, *P *= 0, *D *= 1, and *Q *= 1. Observations were marked as additive outlier if the likelihood ratio test statistic (for an additive outlier) for the observation was below a threshold of -6 [21]. For those observations classified as not available or as a lower additive outlier that were not at the beginning or end of a series, values for the number of malaria positive blood films were estimated through a one-step-ahead SARIMA forecasting model on both the original series and on the reversed series, and the two estimates were averaged. This approach has been discussed by Mwaniki and colleagues [22]. Finally, the MOH area data series were aggregated to district resolution before analysis, as these spatial units remained constant over the study period, whereas for many MOH areas boundaries changed (within district boundaries) over the study period.

### Seasonal adjustment method with last three observations

Abeku and colleagues [16] tested a seasonal adjustment method on malaria data in Ethiopia and found that it performed better in comparison to SARIMA models. They obtained best results when using a three year "training" time series. The prediction formula used is as follows:

$${\hat{z}}_{t}=\frac{1}{3}\sum_{k=1}^{k=3}\left\{z_{t-12k}\right\}=\frac{1}{3}\sum_{l=1}^{l=3}\left\{z_{t-l}-\frac{1}{3}\sum_{k=1}^{k=3}\left\{z_{t-12k-l}\right\}\right\}.$$

### Model evaluation

For each district, model parameters were estimated on approximately the first half of the malaria case time series (January 1972 – December 1987), and one to four step ahead (out of sample) predictions were made on the second half (January 1988 – December 2005) with the parameters fixed.

For selection of the best predictive models, all models tested were evaluated on the prediction criterion which was defined as the mean absolute relative error (*mare*) of back transformed out of sample predictions:

$$(mare)=\frac{1}{N}\sum_{i=1}^{N}\left|\frac{y_{t}-{\hat{y}}_{t}}{y_{t}+1}\right|$$

where ${\hat{y}}_{t}$ is the predicted number of malaria positive cases at time *t*, and *N *is the number of predictions. Predictions needed to be genuinely out of sample in order to prevent bias towards more parameter models. The (*mare*) was used rather than mean square error, as the malaria count time series show widely differing variances across the series [20]. The best model was that with the lowest prediction criterion for a given time series.

## Results

**Figure 2:**
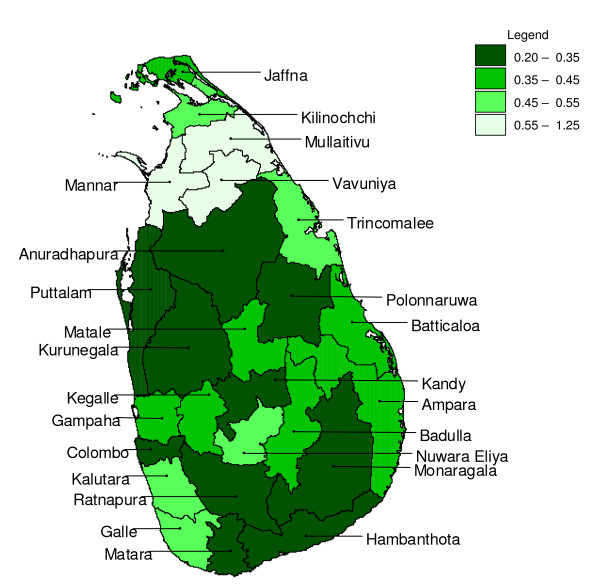
**Mean absolute relative error in districts at a 1 month forecasting horizon**. Mean relative absolute error of out of series prediction at a forecasting horizon of 1 month ahead for districts in Sri Lanka for the best model (without the inclusion of rainfall as a covariate) tested.

The best model (without extrinsic explanatory variables) varied by district and forecasting horizon (Table 1). For instance, for the district of Ampara, for a one month forecasting horizon, the best model was an ARIMA (2,1,1) model with seasonality modelled through a harmonic with a period of one year and a harmonic with a period of six months. For further forecasting horizons, the ARIMA(0,1,2) model with seasonality through a first order seasonal autoregressive and a first order seasonal moving average component was best for the district of Ampara. The best model was most often of the SARIMA class, followed by the class of models modelling seasonality through second order harmonics. For a few districts, at some forecasting horizons, exponential smoothing was best (Table 2). The seasonal adjustment method performed worst (Not shown). The mean relative absolute error of forecasts varied over the districts (for the same forecasting horizon, see Figure 2), and increased with forecasting horizon. The *mare *was relatively high for the districts Galle and Kalutara in the south west, and Nuwara Eliya in the central hill country, which have low malaria endemicity. The *mare *was also (very) high for the districts affected by the armed conflict in the north and east. Within a model class, the most complicated model tested was not necessarily the best model, and often the prediction improvement obtained by fitting an extra (S)ARIMA parameter as compared to more parsimonious models was marginal.

**Table 1:** Mean absolute relative error of out of series prediction at forecasting horizons of 1 to 4 months ahead for districts in Sri Lanka for the best (S)ARIMA model tested.

District	Horizon 1		Horizon 2		Horizon 3		Horizon 4	
	Criterion	Model (pdqPDQ)	Criterion	Model (pdqPDQ)	Criterion	Model (pdqPDQ)	Criterion	Model (pdqPDQ)

Ampara	0.37	012SOH	0.48	012101	0.58	012101	0.60	012101
Anuradhapura	0.23	211101	0.37	210110	0.45	012110	0.51	210110
Badulla	0.43	110SOH	0.62	111SOH	0.75	212101	0.74	112100
Batticaloa	0.36	010011	0.54	012101	0.66	012101	0.78	012101
Colombo	0.35	011000	0.38	112000	0.43	211001	0.46	011000
Galle	0.49	212002	0.58	211101	0.63	211101	0.71	211110
Gampaha	0.40	011111	0.56	011SOH	0.67	011SOH	0.78	011SOH
Hambantota	0.31	010101	0.47	110101	0.60	210101	0.71	210101
Jaffna	0.42	010011	0.58	012111	0.74	012011	0.82	012SOH
Kalutara	0.54	112100	0.72	011000	0.79	110000	0.79	110000
Kandy	0.33	012101	0.43	012101	0.48	112SOH	0.51	212SOH
Kegalle	0.37	010SOH	0.55	211011	0.66	211SOH	0.75	211SOH
Kilinochchi	0.51	010101	0.95	010101	2.13	111010	2.13	010002
Kurunegala	0.25	011011	0.41	010011	0.53	011011	0.63	011011
Mannar	1.16	011100	0.97	012101	1.10	112100	1.18	111101
Matale	0.37	110101	0.53	110101	0.62	212011	0.70	112011
Matara	0.35	212101	0.40	011101	0.46	212101	0.49	0110111
Moneragala	0.29	110100	0.40	011100	0.48	210100	0.56	011100
Mullaitivu	1.03	111100	1.70	112000	2.00	110000	2.58	111SOH
Nuwara Eliya	0.48	212111	0.58	212101	0.66	212101	0.68	111000
Polonnaruwa	0.32	111101	0.47	012101	0.57	111011	0.66	111011
Puttalam	0.35	010101	0.46	010101	0.60	212101	0.72	010101
Ratnapura	0.30	011111	0.43	012111	0.50	210111	0.57	112111
Trincomalee	0.53	112000	0.79	010100	1.05	010100	1.15	112111
Vavuniya	1.22	012000	1.43	012101	1.41	211101	1.48	012101

Legend: pdq = order of autoregressive component, integrated component and moving average component; PDQ = order of seasonal autoregressive component, seasonal integrated component and seasonal moving average component; SOH = seasonality through second order harmonic;

**Table 2:** Mean absolute relative error of out of series prediction at forecasting horizons of 1 to 4 months ahead for districts in Sri Lanka for Holt Winters models.

District	Horizon 1		Horizon 2		Horizon 3		Horizon 4	
	
Model	H	HW	H	HW	H	HW	H	HW
Ampara	0.43	0.39	0.65	0.52	0.83	0.63	0.86	0.67
Anuradhapura	0.34	***0.22***	0.66	***0.35***	0.99	***0.45***	1.22	0.53
Badulla	0.46	0.54	0.67	0.75	0.87	0.95	0.84	0.96
Batticaloa	0.41	0.41	0.65	0.65	0.82	0.82	0.97	0.97
Colombo	***0.35***	0.37	0.39	0.43	0.44	0.48	***0.46***	0.53
Galle	0.50	0.61	0.59	0.74	0.67	0.83	0.79	0.96
Gampaha	0.43	0.43	0.59	0.59	0.70	0.70	0.78	0.78
Hambantota	0.36	0.36	0.57	0.56	0.76	0.73	0.88	0.87
Jaffna	0.43	0.46	0.62	0.63	0.79	0.85	0.85	0.97
Kalutara	0.55	0.61	0.72	0.80	0.81	0.91	0.88	0.97
Kandy	0.37	0.37	0.50	0.50	0.56	0.57	0.57	0.57
Kegalle	0.39	0.40	0.63	0.62	0.83	0.82	0.94	0.95
Kilinochchi	0.58	0.60	1.08	1.12	2.50	2.26	2.70	2.17
Kurunegala	0.34	0.26	0.61	0.43	0.76	0.57	0.85	0.70
Mannar	1.41	1.57	1.74	1.98	1.61	2.63	1.78	2.28
Matale	0.45	0.41	0.73	0.63	0.96	0.74	1.13	0.81
Matara	0.37	0.35	0.42	0.40	0.49	0.48	0.52	0.52
Moneragala	0.31	0.31	0.42	0.41	0.54	0.52	0.62	0.63
Mullaitivu	1.08	1.19	1.73	1.70	2.21	2.54	2.73	***2.38***
Nuwara Eliya	0.49	0.50	0.61	0.60	0.69	0.69	0.69	0.69
Polonnaruwa	0.37	0.37	0.60	0.60	0.76	0.76	0.82	0.82
Puttalam	0.42	0.37	0.67	0.49	0.88	0.64	1.00	0.76
Ratnapura	0.36	0.31	0.55	0.47	0.64	0.56	0.74	0.66
Trincomalee	0.53	0.56	0.82	***0.75***	1.15	***0.97***	1.35	***1.07***
Vavuniya	1.89	2.02	2.82	3.93	2.45	14.21	2.19	4.11

H = Holt's two parameter exponential smoothing; HW = Holt-Winters three parameter exponential smoothing (including seasonality). Values in bold italic represent a better *mare *as compared to the best (S)ARIMA model (without rainfall).

In the analysis of covariates for the mean term, only the (S)ARIMA models shown in Table 1 were tested. Only for the districts Mannar and Ampara, inclusion of malaria in neighbouring districts lowered one month ahead *mare*, with 6.8% and 4.6%, respectively. For many other districts, inclusion of malaria in neighbouring districts raised the *mare*.

Inclusion of a rainfall variable lowered the *mare *with 2.5% or more for eight districts at one or more horizons (Table 3). Logarithmically transformed rainfall lowered the *mare *for Gampaha District (at horizons of three and four months), Mannar District (at a horizon of one month) and Vavuniya District (at a horizon of four months). Logarithmically transformed rainfall with a separate coefficient for each calendar month lowered the *mare *for Ratnapura District (at horizons of three and four months), and Trincomalee District (at horizons of two and three months). Rainfall factored into quintiles (allowing a non-linear relationship) lowered the *mare *for Moneragala District (at a horizon of two months) and Mullaitivu District (at a horizon of one month). The rainy day index lowered the *mare *for Moneragala District (at a horizon of three months), and the rainy day index with a separate coefficient for each calendar month lowered the *mare *for Badulla District at a horizon of four months.

**Table 3:** Districts in Sri Lanka for which inclusion of a covariate in the mean term of the best (S)ARIMA model tested improved the mean absolute relative error of out of series prediction at forecasting horizons of 1 to 4 months ahead.

District	Horizon (months)	Lag (months)	covariate	Improvement (%)
Badulla	4	4	rainy day index, with a separate coefficient for each calendar month	6.5
Gampaha	3	4	logarithmically transformed total monthly rainfall (mm)	3.8
Gampaha	4	4	logarithmically transformed total monthly rainfall (mm)	4.5
Mannar	1	2	logarithmically transformed total monthly rainfall (mm)	5.2
Moneragala	2	2	monthly rainfall factored into quintiles	4.1
Moneragala	2	3	rainy day index	4.6
Moneragala	3	3	rainy day index	3.2
Mullaitivu	1	1	monthly rainfall factored into quintiles	2.6
			logarithmically transformed total monthly rainfall (mm), with a separate	
Ratnapura	3	4	coefficient for each calendar month	3.9
			logarithmically transformed total monthly rainfall (mm), with a separate	
Ratnapura	4	4	coefficient for each calendar month	3.6
			logarithmically transformed total monthly rainfall (mm), with a separate	
Trincomalee	2	2	coefficient for each calendar month	8.4
			logarithmically transformed total monthly rainfall (mm), with a separate	
Trincomalee	3	3	coefficient for each calendar month	9.2
Vavuniya	4	4	logarithmically transformed total monthly rainfall (mm)	2.5

## Discussion

### Models without extrinsic explanatory variables

The mean absolute relative prediction error calculated by the best model (without extrinsic explanatory variables) tested was quite large for many districts. However, as the models were fitted to only half of the length of the time-series available for the purpose of model testing (out of sample predictions are required), it is expected that for application in a forecasting system where the full series are used for fitting, the error will be reduced. For some districts in the north, the forecasting error was particularly large. For these districts, a relatively large proportion of the data had been imputed, and the quality of the existing data is likely compromised by the armed conflict in this region. General issues related to data quality are discussed elsewhere [23]. In this (primarily) temporal study, issues relating to spatial variation in data quality (*e.g*. through access to health facilities for sampling) are of less importance than those pertaining to temporal aspects, such as the deployment of mobile clinics during specific periods. Despite the sometimes large prediction errors, especially for larger forecasting horizons, prediction intervals yielded by these models (albeit less accurate for low predicted mean counts due to the Gaussian approximation used) could aid the AMC in assessing the risk of malaria in the near future, and adjust resources for preparedness accordingly. Although the best model selected varied among districts and over forecasting horizons, the difference between models was often small. Instead of specifying a different model for each situation, for practical implementation, it may be worth selecting for each district (or even group of districts) one model that performs well on average over a range of forecasting horizons (and districts within the group), provided that the prediction quality does not deteriorate more than a set percentage. A pilot forecasting system using district specific SARIMA models is currently being tested by the AMC in Sri Lanka (the system currently uses models without explanatory variables because a system to incorporate newly observed data is not yet in place). As the spatial resolution of the forecasting models presented is at district level, predictions will not help spatially targeted control at sub district level. For this, regional malaria control officers will have to rely on their experience of where within the district cases tend to occur if they occur, possibly aided by existing malaria maps at sub district level [23].

### Models including rainfall as explanatory variable

It should be kept in mind that the malaria count data are not a direct proxy of new malaria infections or even infective bites. Recrudescent and relapsing cases (mostly due to ineffective drugs) occur multiple times in the malaria dataset, and immunity may play a role during periods of higher endemicity, thus weakening explanatory power of a variable such as rainfall, which would probably be better at explaining infectious bites [24].

Rainfall improved the prediction for eight districts, at one or more of the tested forecasting horizons, but it also worsened the prediction for other districts at various horizons. For some districts, the rainfall preceding malaria by two months improved performance at forecasting horizons of three and four months (not shown), but this is of little use in a forecasting system, unless rainfall can be predicted with high confidence (one to two months ahead). Although only tested for four districts, the rainy day index was promising as it improved results for two out of these.

The results of the analysis incorporating rainfall was remarkably different from what would be expected based on results of cross correlation analysis of malaria and rainfall. Although both (pre-whitened) cross correlations [25] and the improvements of rainfall to prediction accuracy were generally low, (pre-whitened) cross correlation analysis indicated that prediction accuracy for a number of districts in the centre-west and centre-south of the country was likely to benefit from rainfall (with a single, negative coefficient), and the present analysis found such improvement only for Gampaha District.

Varying seasonal effects of rainfall on malaria in seasonal inter-annual analysis [25] suggested that especially for districts in the north and east, prediction models would benefit from addition of rainfall with a monthly varying coefficient. However, only for the districts Trincomalee and Ratnapura (the latter situated in the south), this was the case. It is possible that for most of the districts, the training time series was too short (sixteen years) to estimate each of the twelve coefficients reliably.

For most districts (except the districts Mannar, Mullaitivu and Vavuniya), presumably due to strong short term temporal auto correlation, the observed number of malaria cases is a good predictor at a one month forecasting horizon (Figure 2). Interestingly, for Mannar and Mullaitivu, rainfall could improve predictions somewhat at this horizon. For the remaining six districts for which rainfall was found to improve predictions, this was mostly at larger forecasting horizons. For prediction with an even further forecasting horizon, use of predicted rainfall might be feasible. Rainfall could be predicted up to several months ahead using, for instance, the El Niño southern oscillation index, which in itself has proven to have a statistically significant relationship with malaria epidemics in the pre control era [6]. During periods when case reporting might be compromised (*e.g*. through civil war), rainfall may gain in relative importance as a predictor of the true number of cases, although this would be difficult to validate. It should be noted that high cross correlations between (not prewhitened, seasonal) malaria time series and extrinsic covariates (with seasonality) such as rainfall and temperature may exist [25], and these covariates may perform well in models that do not include fixed seasonal or auto-regressive seasonal terms, but not necessarily better than models that do include fixed seasonal or auto-regressive seasonal terms.

The contribution of rainfall to reducing the prediction error was modest, and it is arguable whether or not the achieved reduction in the prediction error (for instance, a reduction of 9.2%, bringing the mean relative error down from 1.05 to 0.95 at a horizon of three months in the district Trincomalee) merits investing in a system where at the end of each month, monitored rainfall data are collected and processed to obtain average values for the district surface for entering into the prediction model. However, as such data become increasingly available with the development of monthly rainfall data monitoring systems at district and sub district scale [26], the reduction in prediction error gained might well outweigh the (reduced) effort.

The modest improvement found by including rainfall compares well to a study in Ethiopia [17]. This study also found modest improvement (a reduction of 10.7% in the number of one step ahead predictions that were more than twice or less than half the observed value) by a model incorporating rainfall and temperature over a simple ARIMA(0,1,0) model that uses the previous value as predictor. However, the improvement would probably have been less if it had been tested on out of sample predictions. It is interesting to note that in this same paper, Abeku and colleagues [17] found that the ARIMA(0,1,0) performed better than the seasonal adjustment method (which performed unsatisfactorily on Sri Lankan data), whereas previously the seasonal adjustment method had been reported as superior [16]. Another study carried out in Ethiopia found that a prediction model including rainfall, temperature and cases in the previous month performed well in flagging potential epidemics as compared to observed cases, but was not compared with a prediction model based on past cases alone [13], therefore, the relative improvement cannot be assessed and compared to the improvement found in the present study.

### Some explanatory variables not considered in this study and suggestions for future work

Other rainfall related variables such as soil moisture content and river flow might give better results than observed rainfall, as these are more directly related to malaria vector breeding conditions. The main malaria vector in Sri Lanka, *Anopheles culicificacies*, primarily breeds in pooling rivers (although there are also other habitats such as those mentioned above). In general, rainfall and river flow show strong cross correlations, and therefore rainfall can serve as a proxy for river flow. However, under dryer conditions -important for vector breeding- direct runoff will be decreased and river flow will be increasingly influenced by other hydrological processes such as percolation and evapo-transpiration. As river flow and soil moisture are difficult to measure over large surfaces (and long term data with good spatial resolution are not readily available), they can be estimated through modelling, although rainfall will remain an important variable in such models [27]. Vegetation indices such as the normalized difference vegetation index (NDVI) were not studied because it can be argued that in a primarily temporal analysis, observed station rainfall is more closely related to breeding conditions than temporal changes in NDVI. Furthermore, remote sensing images of Sri Lanka suffer from obstruction by cloud cover during the monsoons. Apart from rainfall and rainfall related variables, another variable that is expected to have a strong temporal effect on malaria is vector control. This variable was not taken into account due to incomplete data. The effect of rainfall on malaria could be studied for data in the pre-control era [6], but with the reality of control measures being carried out this will be of little use for current prediction. The primary goal of this study was to develop an easy to use practical prediction model with the data available. The effect of missing important variables in a prediction model will not invalidate results, but will be expressed in larger prediction intervals. Nevertheless, inclusion of a malaria control variable, at least for those districts with relatively complete data, merits investigation.

Temperature was not considered as part of the present analysis, based on the assumption that it was of less importance than rainfall, showing little temporal variability (because Sri Lanka is situated close to the equator), and a large part of its temporal variability being governed by rainfall. Except for the hill country (covering largely the districts Nuwara Eliya, Kandy and Badulla), the temperature is generally conducive to malaria transmission throughout the year. However, for parts of the districts Kandy and Badulla, temperature could be limiting during part of (but not the whole of) the year and its role in these districts merits further investigation. In a study on the relationship between malaria and rainfall and temperature in Ethiopia, rainfall was found to be important in hot districts which were situated below 1650 m, but not in cold districts above 1650 m, where minimum temperature was significant [28]. Other environmental factors that are often considered in malaria studies are altitude and land use. Altitude was not taken into account because it does not fluctuate over time, and is thus of no use for temporal forecasting. Land use databases with monthly temporal resolution were not available. The performance of rainfall over several lag times accumulated, or at thresholds, remains to be explored. It is tempting to try to build a space-time malaria forecasting model for the whole of Sri Lanka with more statistical power than a separate model for each district. Such a model should allow for regionally varying functions of covariates and take into account spatial auto correlation between districts or even MOH areas. For the purpose of a forecasting system, with the presently small malaria counts in most districts, it might be more appropriate to model the malaria counts directly following a negative binomial distribution, rather than through a logarithmic transformation [29]. Although easy to implement (and used in this work as well as work by others), the Gaussian (Normal) approximation of the malaria count data (after logarithmic transformation) is likely to affect the accuracy of the predictions, particularly when the counts are expected to be close to zero. Most importantly, this may yield inaccurate prediction intervals during periods of low case numbers. Although not so important in the current study (where models were evaluated on the means of the predictions), prediction intervals are very important for control planning.

## Authors' contributions

OJTB conceptualized and conceived of the analysis, performed the data treatment and analysis, and drafted the manuscript. PV participated in the conceptualization, edited the manuscript and critically revised the statistical methodology. DMG participated in the conceptualization of the study and edited the manuscript. GNLG provided the data and helped define the scope of the paper. PHA participated in defining the approach to analysis, edited and critically reviewed the paper for intellectual content. All authors read and approved of the manuscript.
